# Novel [^99m^TcN]^2+^ Labeled EGFR Inhibitors as Potential Radiotracers for Single Photon Emission Computed Tomography (SPECT) Tumor Imaging

**DOI:** 10.3390/molecules19055508

**Published:** 2014-04-29

**Authors:** Mingxia Zhao, Hongyu Ning, Man Feng, Shilei Li, Jin Chang, Chuanmin Qi

**Affiliations:** Key Laboratory of Radiopharmaceuticals, Ministry of Education, College of Chemistry, Beijing Normal University, Beijing 100875, China

**Keywords:** [^99m^TcN]^2+^ complex, 4-aminoquinazoline, EGFR, tumor imaging

## Abstract

The epidermal growth factor receptor (EGFR) is overexpressed in many cancers, including breast, ovarian, endometrial and non-small cell lung cancer. An EGFR-specific imaging agent could facilitate clinical evaluation of primary tumors or metastases. To achieve this goal, 4-(2-aminoethylamino)-6,7-dimethoxyquinazoline (ADMQ) was synthesized based on a 4-aminoquinazoline core and then conjugated with *N*-mercapto- acetylglycine (MAG) and *N*-mercaptoacetyltriglycine (MAG_3_), respectively, to give compounds **1** and **2**. The final complexes **[^99m^TcN]-1** and **[^99m^TcN]-2** were successfully obtained with radiochemical purities of >99% and >98% as measured by radio-HPLC. No decomposition of the two complexes at room temperature was observed over a period of 2 h. Their partition coefficients indicated they were hydrophilic and the electrophoresis results showed they were negatively charged. Biodistribution in tumor-bearing mice demonstrated that the two new complexes showed tumor accumulation, high tumor-tomuscle (T/M) ratios and fast clearance from blood and muscle. Between the two compounds, the ^99m^TcN-MAG_3_-ADMQ (**[^99m^TcN]-2**) showed the better characteristics, with the tumor/muscle and tumor/blood ratios reached 2.11 and 1.90 at 60 min post-injection, 4.20 and 1.10 at 120 min post-injection, suggesting it could be a promising radiotracer for SPECT tumor imaging.

## 1. Introduction

Epidermal growth factor receptor (EGFR) is a member of the epidermal growth factor (EGF) family of tyrosine kinase receptors (which also includes ErbB2, ErbB3 and ErbB4). Ligand binding to the extracellular domain of the receptor results in the activation of the receptor [1,2]. The activated receptor can dimerize with other EGFRs followed by phosphorylation of tyrosine residues on the receptors. This active conformation makes it possible for TK domain of the receptors both to bind ATP and to transphosphorylate each other [3,4]. Receptor autophosphorylation on tyrosine residues both enhances the activity of kinases and provides docking sites for downstream signal transduction molecules that habor SH2 or PTB domains. These interaction activate signal transduction pathways, which ultimately lead to multiple cellular processes, such as proliferation, differentiation, apotosis, angiogenesis, cell adhesion and movement [5,6,7].

Correlation between EGFR overexpression and metastasis foramation, therapy resistance, poor prognosis, and short survival has prompted the design and development of various anti-EGFR-targeted therapies [8,9,10,11,12]. Several quinazoline derivatives have been used as tyrosine kinase inhibitors targeting EGFR, such as gefitinib, erlotinib and lapatinib [13,14,15,16]. However, analysis of the expression of EGFR and the presence of mutations requires a tumor biopsy, which is not possible to get in all situations [17,18,19,20]. Thus, the ability to noninavasively quantitate EGFR content in tumors would aid in selecting patients who are likely to benefit from anti-EGFR-targeted therapy and in monitoring such treatment. Therefore, there has been a growing interest in the use of EGFR-TK inhibitors as radiotracers for molecular imaging of EGFR overexpressing tumors via nuclear medicine modalities such as SPCET and PET [21,22,23,24,25].

The [^99m^TcN]^2+^ core exhibits a very high chemical stability and high affinity toward chelating ligands containing sulfur atoms which when present in the molecular structure of a radiopharmaceutical may dramatically affect its physical and biological behaviour [23,26]. Based on the good performance of quinazoline derivatives in anticancer applications, we have designed and synthesized 4-(2-aminoethyl- amino)-6,7-dimethoxyquinazoline (ADMQ, **8**) by introducing a solubilizing basic ethylenediamine moiety at the 4-position of 4-chloro-6, 7-dimethoxyquinazoline (**7**) to link to the target molecules with MAG or MAG_3_, thus affording a new N_3_S type ligand. So far, the use of the ADMQ moiety **8** in the preparation of ^99m^TcN complexes as targeted agents for tumor imaging has not been reported. Herein, ^99m^TcN-MAG-ADMQ (**[^99m^TcN]-1**) and ^99m^TcN-MAG_3_-ADMQ (**[^99m^TcN]-2**) were designed and synthesized to evaluate the feasibility of the ^99m^Tc-labeled 4-aminoquinazoline derivatives as useful candidates for tumor imaging.

## 2. Results and Discussion

### 2.1. Chemistry

**Scheme 1:**
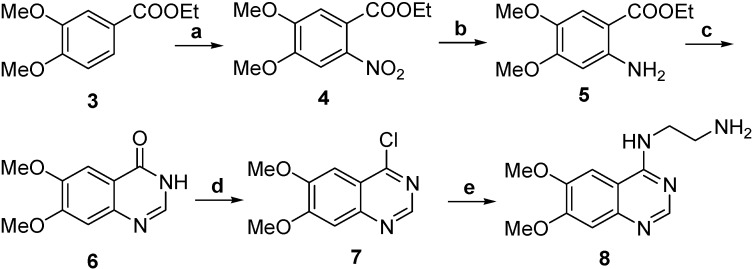
Synthesis of ADMQ.

**Scheme 2:**
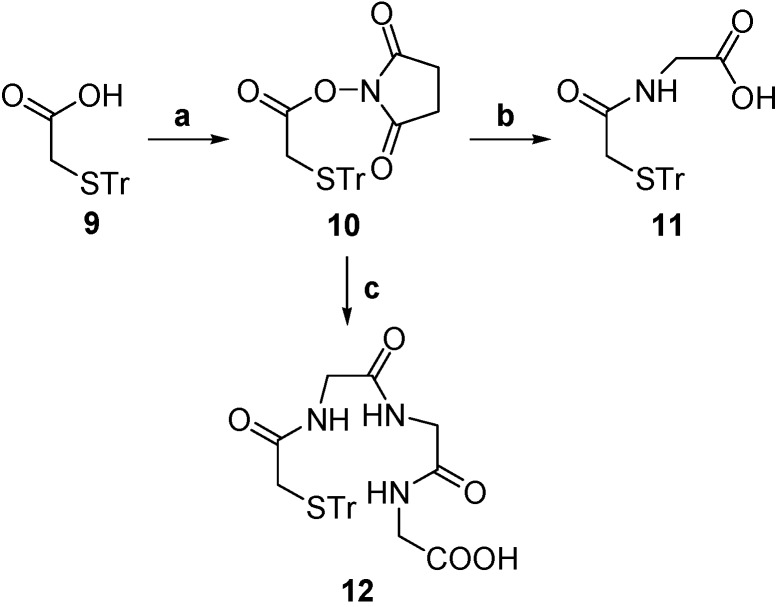
Synthesis of Tr-MAG and Tr-MAG_3_.

The synthesis of 4-(2-aminoethylamino)-6,7-dimethoxyquinazoline was carried out using the procedure shown in Scheme 1. A solution of compound **3** in AcOH was added to nitric acid solution under stirring at 0–5 °C for 30 min and then at room temperature for 24 h to obtain **4**. Compound **4** was hydrogenated with Pd/C in methanol at room temperature to obtain **5**. Compound **5** was reacted with formamide at 165–170 °C under N_2_ to obtain **6**, which was treated with thionyl chloride and DMF to get chloro derivative **7** and then **7** coupled with ethylenediamine in *i*-PrOH at 80 °C to obtain **8**. The synthesis of compounds **9**–**12** is shown in Scheme 2. Compound **9** in which the thiol group was protected by trityl chloride in advance was reacted with N-hydroxysuccinimide (NHS) using dicyclohexylcarbodiimide (DCC) as condensation reagent to obtain the active ester **10**. The active ester **10** was reacted with the amine group of glycine to provide the Tr-MAG (**11**).

*Reagents and conditions:* (a) AcOH, HNO_3_, 0–5 °C to rt, 24 h, 90%; (b) Pd/C, H_2_, rt, 8 h, 86%; (c) HCONH_2_, 165–170 °C, 6 h, 73%; (d) SOCl_2_, DMF, reflux, 4 h, 82%; (e) NH_2_CH_2_CH_2_NH_2_, *i*-PrOH, 80 °C, 4 h, 81%.

*Reagents and conditions:* (a) NHS, DCC, anhydrous THF, −5 °C to rt, 10 h, 82%; (b) CH_3_CN, glycine, 50–60 °C, 2 h, 78%; (c) CH_3_CN, triglycine, 50–60 °C, 2 h, 71%.

Tr-MAG_3_ (**12**) was synthesized by the same procedure as **11**, except using triglycine instead of glycine. Tr-MAG (**11**) was conjugated with compound **8** with DCC as condensation agent and 4-dimethylaminopyridine (DMAP) as nucleophilic agent to provide the compound **13**. Tr-MAG_3_ (**12**) was conjugated with compound **8** under the same conditions to give compound **14**. Next, the thiol groups were deprotected in trifluoroacetic acid (TFA) to obtain **1**. Compound **2** was prepared using the same method as product **1**.

### 2.2. Radiochemistry

#### 2.2.1. Synthesis of ^99m^TcN-MAG-ADMQ and ^99m^TcN-MAG_3_-ADMQ

**Scheme 3:**
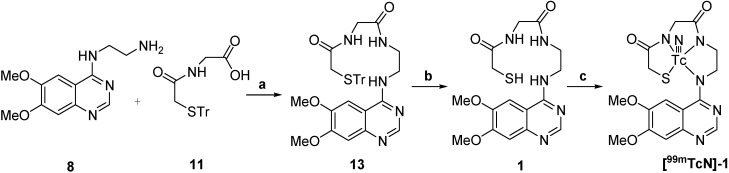
Synthesis of ^99m^TcN-MAG-ADMQ.

**Scheme 4:**
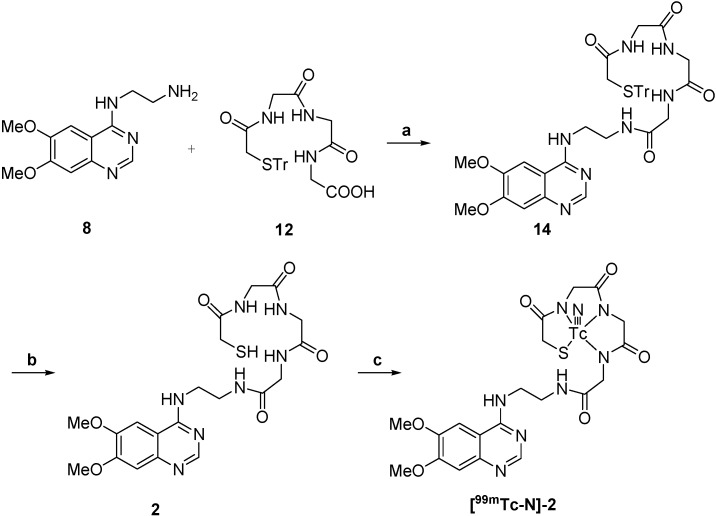
Synthesis of ^99m^TcN-MAG_3_-ADMQ.

Synthesis of the two compounds **[^99m^TcN]-1** and **[^99m^TcN]-2** was performed according to the procedure shown in Scheme 3 and Scheme 4.

*Reagents and conditions:* (a) HOBt, DCC, Et_3_N, anhydrous CH_2_Cl_2_, 0 °C to rt, 10 h, 61%; (b) Et_3_SiH, TFA, rt, 5 min; (c) ^99m^Tc-SDC, SnCl_2_, PDTA, rt to 100 °C, 30 min.

*Reagents and conditions:* (a) HOBt, DCC, Et_3_N, anhydrous CH_2_Cl_2_, 0 °C to rt, 10 h, 60%; (b) Et_3_SiH, TFA, rt, 5 min; (c) ^99m^Tc-SDC, SnCl_2_, PDTA, rt to 100 °C, 30 min.

**Figure 1:**
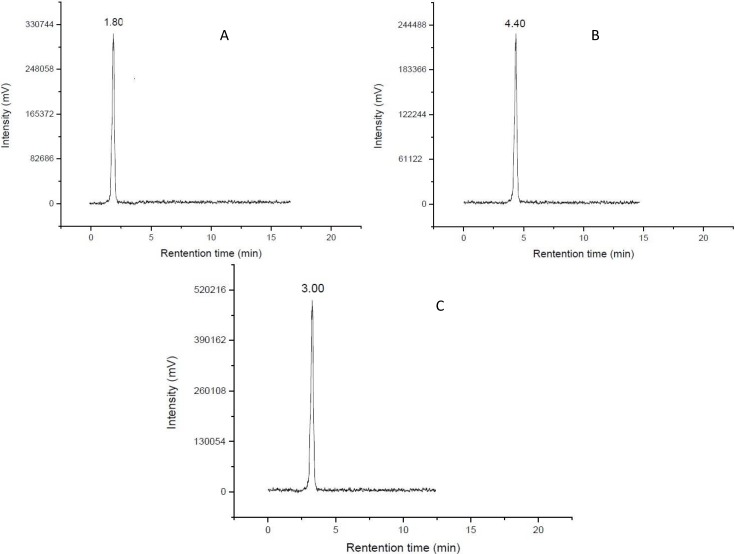
Radio-HPLC chromatograms.

For labeling, **[^99m^TcN]-1** was prepared through a SDH kit. [^99m^TcO_4_]^−^ reacted with SDH in the presence of stannous chloride as reducing agent to form a technetium-99m nitrido intermediate. The [^99m^TcN]^2+^ is a suitable substrate for the substitution reaction with compound **1** at 100 °C for 15 min to give the final complex **[^99m^TcN]-1**. The preparation of **[^99m^TcN]-2** was analogous to that for **[^99m^TcN]-1** given above, except that the ligand used was compound **2**. Early experimental anionic compounds such as [^99g^TcO(MAG_3_)]^−^, [^99g^TcO(MAG_3_OMe)] and [ReO(MAG_2_-pABAH)]^−^ established the structure model using the N_3_S ligand system [27,28]. It is reasonable to suppose that the structure of the two complexes in this paper might be similar to those that have been reported in the previous work for the same N_3_S ligand system was employed to coordinate to ^99m^Tc. The radiochemical purity of the two complexes was routinely checked by radio-HPLC. The HPLC patterns of **[^99m^TcN]-1** and **[^99m^TcN]-2** are shown in Figure 1. It was observed that the retention time of [^99m^TcN]^2+^_int_ was 1.8 min, while those of **[^99m^TcN]-1** and **[^99m^TcN]-2** were found to be 4.4 min and 3.0 min, respectively. The mean radiochemical purity of the two products was over 90% immediately after the preparation.

(A) ^99m^TcN^2+^, t_R_ = 1.8 min; (B) [^99m^TcN]-1, t_R_ = 4.4 min; (C) [^99m^TcN]-2, t_R_ = 3.0 min.

#### 2.2.2. *In Vitro* Stability

The HPLC analysis results for the two complexes indicated that they were all stable in PBS after incubation for 2 h.

#### 2.2.3. Paper Electrophoresis

Paper electrophoresis showed that about 90% of the initial activity remained in the positive electrode, indicating that they are negatively charged complexes.

#### 2.2.4. Partition coefficients

As far as passive diffusion into tissues and cells is concerned, the lipophilicity of the molecule (generally denoted by log P) should be sufficiently high to allow penetration through the cell membrane. However, high log P values usually lead to slow clearance from blood, accumulation in metabolic tissue, and non-specific binding in tumors. The partition coefficients (log P) of **[^99m^TcN]-1** and **[^99m^TcN]-2** were measured according to the published method [29] (Table 1) and indicated that both complexes were hydrophilic. **[^99m^TcN]-2** was less hydrophilic than **[^99m^TcN]-1**.

**Table 1:** Partition coefficients of **[^99m^TcN]-1** and **[^99m^TcN]-2**.

	[^99m^TcN]-1	[^99m^TcN]-2
log P	−1.81 ± 0.02	−1.57 ± 0.01

#### 2.2.5. Biological Evaluation

Mice were sacrificed and major organs such as brain, liver, lungs, kidneys, spleen, stomach and heart, as well as the tumors and blood, were isolated to evaluate the tissue distribution of the two complexes. The biodistribution results are summarized in Table 2 and Table 3.

**Table 2:** Biodistribution of **[^99m^TcN]-1** in mice bearing S180 tumor (%ID/g) ^a^.

Tissue	30 min	60 min	120 min
Heart	0.27 ± 0.02	0.11 ± 0.01	0.05 ± 0.01
Liver	2.71 ± 0.16	1.01 ± 0.08	0.55 ± 0.11
Spleen	0.31 ± 0.03	0.15 ± 0.01	0.07 ± 0.02
Lung	1.36 ± 0.13	0.94 ± 0.06	0.44 ± 0.09
Kidney	2.42 ± 0.10	2.3 ± 0.07	1.35 ± 0.11
Muscle	0.27 ± 0.01	0.07 ± 0.01	0.06 ± 0.02
Bone	0.19 ± 0.02	0.16 ± 0.04	0.04 ± 0.01
Large Intestine	0.71 ± 0.22	0.79 ± 0.12	0.51 ± 0.10
Small Intestine	0.31 ± 0.01	0.25 ± 0.03	0.18 ± 0.03
Stomach	0.43 ± 0.05	0.04 ± 0.01	0.07 ± 0.03
Brain	0.04 ± 0.01	0.03 ± 0.02	0.01 ± 0.01
Tumor	0.35 ± 0.03	0.31 ± 0.04	0.07 ± 0.01
Blood	1.09 ± 0.11	0.82 ± 0.03	0.11 ± 0.04
T/M ratio	1.29 ± 0.14	1.82 ± 0.33	1.17 ± 0.13
T/B ratio	1.84 ± 0.07	1.94 ± 0.24	1.75 ± 0.16

T/M = tumor-to-muscle, T/B = tumor-to-blood; ^a^ All data are the mean percentage (*n* = 3) of the injected dose of [^99m^TcN]-1; per gram of tissue, ± the standard deviation of the mean.

There were apparent similarities in the biodistribution patterns of the two compounds that demonstrated tumor accumulation, high tumor-to-muscle (T/M) ratio, tumor-to-blood (T/B) ratio and rapid washout from blood. Early hepatic and renal activity reflected the two compounds were excreted through the hepatobiliary as well as the renal system. The clearance rate in the tumor was slower than in other tissues or organs with time, so the activity in the tumor exceeded than that in most other tissue or organs. For **[^99m^TcN]-1**, the tumor-to-muscle (T/M) and tumor-to-blood (T/B) ratios reached 1.82 and 1.94 at 60 min, 1.17 and 1.75 at 120 min post-injection, respectively. For **[^99m^TcN]-2**, the tumor-to-muscle (T/M) and tumor-to-blood (T/B) ratios reached 2.11 and 1.90 at 60 min, 4.20 and 1.10 at 120 min, respectively. Between them, **[^99m^TcN]-2** showed the better characteristics with the higher tumor/muscle ratio that reached to 4.2 at 120 min. The two compounds were hydrophilic so that they were unable to cross the blood brain barrier, thus making their brain uptake much lower. It is deemed worthwhile to modify the structure of ADMQ suitably to render it less hydrophilic to enhance the tumor uptake of its ^99m^Tc labeled complex.

**Table 3:** Biodistribution of **[^99m^TcN]-2** in mice bearing S180 tumor (%ID/g) ^b^.

Tissue	30 min	60 min	120 min
Heart	0.40 ± 0.05	0.12 ± 0.03	0.08 ± 0.01
Liver	0.78 ± 0.09	0.79 ± 0.04	0.39 ± 0.05
Spleen	0.16 ± 0.01	0.08 ± 0.02	0.05 ± 0.01
Lung	0.50 ± 0.11	0.30 ± 0.08	0.14 ± 0.04
Kidney	1.64 ± 0.15	1.97 ± 0.21	1.65 ± 0.16
Muscle	0.25 ± 0.06	0.19 ± 0.02	0.05 ± 0.02
Bone	0.55 ± 0.15	0.21 ± 0.06	0.20 ± 0.01
Large Intestine	0.39 ± 0.17	0.51 ± 0.04	1.22 ± 0.24
Small Intestine	0.99 ± 0.14	0.56 ± 0.09	0.20 ± 0.05
Stomach	0.15 ± 0.03	0.14 ± 0.07	0.04 ± 0.02
Brain	0.02 ± 0.01	0.01 ± 0.01	0.01 ± 0.005
Tumor	0.21 ± 0.07	0.40 ± 0.05	0.21 ± 0.08
Blood	0.96 ± 0.23	0.87 ± 0.16	0.13 ± 0.06
T/M ratio	0.84 ± 0.09	2.11 ± 0.18	4.20 ± 0.25
T/B ratio	0.38 ± 0.05	1.90 ± 0.16	1.10 ± 0.08

T/M = tumor-to-muscle, T/B = tumor-to-blood; ^b^ All data are the mean percentage (*n* = 3) of the injected dose of [^99m^TcN]-2; per gram of tissue, ± the standard deviation of the mean.

## 3. Experimental

### 3.1. General Information

Succinic dihydrazide (SDH) kit was obtained from BeijingShihong Pharmaceutical Center, Beijing Normal University (Beijing, China). ^99^Mo/^99m^Tc generator was obtained from the China Institute of Atomic Energy (CIAE) (Beijing, China). THF was refluxed over sodium/benzophenone and CH_2_Cl_2_ was refluxed over phosphorous CaH_2_. Other solvents were purchased as anhydrous. ^1^H- and ^13^C-NMR spectra were recorded on a Bruker model (Bruker, Karlsruhe, Germany) spectrometer operating in DMSO-*d*_6_ or CDCl_3_ at 400 MHz and 100 MHz, respectively. ^1^H signals were reported in ppm. The IR spectra were recorded using KBr pellets in the 4,000-400 cm^−1^ region on a Nicolet-AVATAR 360 FT-IR spectrometer (Nicolet-AVATAR, Belmont, MA, USA). ESI-MS spectra were obtained on Waters LCT Premier XE (Waters, Milford, MA, USA). HPLC analyses were performed on a Shimadzu SCL-10 AVP (Shimadzu, Kyoto, Japan) equipped with a Packard 500 TR series flow scintillation analyzer (Shimadzu) A C-18 reversed-phase Alltima column (5 μm, 150 mm × 4.6 mm) was used for radiochemical purity analysis.

### 3.2. Synthesis

The preparation of compounds **3**–**8** was carried out by the similar procedure described in [30,31] with some modifications. The synthesis of compounds **9**–**12** was carried out by the same procedure described in [32]. The preparation and the analysis data of the compounds are shown below.

*Ethyl 4,5-dimethoxy-2-nitrobenzoate* (**4**). A solution of ethyl 3,4-dimethoxybenoate (6.0 g, 0.026 mol) in AcOH (23 mL) was added dropwise to nitric acid (6 mL, 65%) at 0–5 °C, and stirred at the same temperature for 30 min and then for 24 h at room temperature. Reaction progress was monitored by TLC and it was found to be complete after this time. The reaction mixture was poured onto ice/water to afford the yellow precipitate which was filtered and washed with ice water. The precipitate dried over P_2_O_5_ to afford the product **4** (5.91 g, yield 90.3%), m.p.: 78–79 °C.; IR (cm^−1^): ν 3451, 2983, 1722, 1609, 1536, 1389, 1361, 1294, 1219, 1016, 877; ^1^H-NMR (CDCl_3_) δ (ppm): 7.46 (1H, s, 6-H), 7.08 (1H, s, 3-H), 4.39 (2H, q, *J* = 7.08 Hz, -CH_2_), 3.98 (6H, s, -OCH_3_) 1.35 (3H, t, *J* = 7.16 Hz, -CH_3_). ^13^C-NMR (CDCl_3_) δ (ppm): 165.78 (C=O), 152.50 (5-C), 150.33 (4-C), 141.25 (2-C), 122.03 (1-C), 110.85 (6-C), 106.98 (3-C), 62.42 (-CH_2_), 56.61 (-OCH_3_), 56.57 (-OCH_3_), 13.74 (-CH_3_).

*Ethyl 2-amino-4,5-dimethoxybenzoate* (**5**). A solution of **4** (5.0 g, 0.020 mol) in methanol (30 mL) was hydrogenated with Pd/C (1.15 g) at room temperature. Reaction development was monitored by TLC and the reduction continued until no more hydrogen was consumed. The catalyst was filtered off and methanol removed to afford a brown precipitate **5** which was filtered through a glass funnel and dried under reduced pressure (3.88 g, yield 85.5%), m.p.: 76–78 °C. IR (cm^−1^): ν 3489, 3375, 2978, 2829, 1680, 1591, 1516, 1398, 1300, 1157, 1029, 785, 592; ^1^H-NMR (CDCl_3_) δ (ppm): 7.32 (1H, s, 6-H), 6.14 (1H, s, 3-H), 5.57 (2H, br, -NH_2_), 4.32 (2H, q, *J* = 7.08 Hz, -CH_2_), 3.87 (3H, s, -OCH_3_), 3.87 (3H, s, -OCH_3_), 1.38 (3H, t, *J* = 7.12 Hz, -CH_3_). ^13^C-NMR (CDCl_3_) δ (ppm): 167.79 (C=O), 154.85 (4-C), 147.06 (2-C), 140.64 (5-C), 113.06 (6-C), 102.52 (1-C), 99.46 (3-C), 60.09 (-CH_2_), 56.55 (-OCH_3_), 55.76 (-OCH_3_), 14.50 (-CH_3_).

*6,7-Dimethoxyquinazoline-4-one* (**6**). A solution of **5** (2.5 g, 0.011 mol) in formamide (50 mL) was heated to 165–170 °C under N_2_ for 6 h. When TLC indicated the absence of starting material, the reaction mixture was cooled and the amber sticky precipitate was filtered using a sintered glass frit and dried on the air (1.90 g, yield 73.2%), m.p.: 295–296 °C. IR (cm^−1^): ν 3159, 3015, 2843, 2768, 1658, 1610, 1504, 1439, 1357, 1271, 1078, 877, 642; ^1^H-NMR (DMSO-*d*_6_) δ (ppm): 12.03 (1H, s, -NH), 7.99 (1H, s, 2-H), 7.42 (1H, s, 5-H), 7.11 (1H, s, 8-H), 3.88(3H, s, -OCH_3_), 3.85 (3H, s, -OCH_3_); ^13^C-NMR (DMSO-*d*_6_) δ (ppm): 159.99 (4-C), 154.43 (7-C), 148.53 (6-C), 144.84 (2-C) 143.77 (9-C), 115.58 (10-C), 108.00 (5-C), 104.93 (8-C), 55.88 (-OCH_3_), 55.66 (-OCH_3_).

*4-Chloro-6, 7-dimethoxyquinazoline* (**7**). A stirred mixture of **6** (2.0 g, 0.010 mol), thionyl chloride (30 mL) and *N*,*N-*dimethylformamide (0.6 mL) was heated under reflux for 4 h. The solvent was removed in vacuo to obtain the off-white crude product and the crude product was recrystallized in DMF to obtain the compound **7** (1.81g, yield 81.7%), m.p.: 178 °C. IR (cm^−1^): ν 3431, 1618, 1560, 1508, 1412, 1348, 1234, 1161, 968, 850, 698; ^1^H-NMR (DMSO-*d*_6_) δ (ppm): 8.88(1H, s, 2-H), 7.46(1H, s, 5-H), 7.41 (1H, s, 8-H), 4.00 (6H, s, -OCH_3_); ^13^C-NMR (DMSO-*d*_6_) δ (ppm): 158.82 (2-C), 155.05 (7-C), 149.34 (6-C), 145.86 (9-C), 138.85 (10-C), 114.73 (5-C), 105.52 (8-C), 104.11 (C-4), 56.16 (-OCH_3_), 55.94 (-OCH_3_).

*4-(2-Aminoethylamino)-6, 7-dimethoxyquinazoline* (ADMQ, **8**). To a solution of **7** (1.0 g, 4.5 mmol) in *i*-PrOH (30 mL) ethylenediamine (0.45 mL, 6.75 mmol) was added. The reaction mixture was heated to 80 °C and stirred for 4 h and then was quenched in ice water and filtered to afford the solid product **8** (0.9 g, yield 80.6%). IR (cm^−1^): ν 3504, 3419, 3340, 2986, 2071, 1625, 1598, 1541, 1511, 1421, 1251, 843; ^1^H-NMR (DMSO-*d*_6_) δ (ppm): 8.37 (1H, s, 2-H), 8.21 (1H, s, -NH), 7.67(1H, s, 5-H), 7.11(1H, s, 8-H), 3.90 (6H, s, -OCH_3_), 3.75 (2H, t, *J* = 5.96 Hz, -CH_2_), 3.09 (2H, t, *J* = 5.96 Hz, -CH_2_); ^13^C-NMR (DMSO-*d*_6_) δ (ppm): 158.34 (7-C), 153.82 4-C), 153.13 (2-C), 148.30 (6-C), 145.97 (9-C), 108.60 (10-C), 106.90 (8-C), 102.48 (5-C), 56.18 (-OCH_3_), 55.61 (-OCH_3_), 38.50 (2CH_2_); MS (ESI): M = 248.1 (Found: 247.2); Anal. Calcd. for C_12_H_16_N_4_O_2_: C, 58.05; H, 6.50; N, 22.57%. Found: C, 57.88; H, 5.92; N, 22.62%.

*Conjugation of Tr-MAG and ADMQ* (Tr-MAG-ADMQ, **13**). Triethylamine (0.13 mL, 0.9 mmol ) was added dropwise into a solution of **8** (148 mg, 0.6 mmol) in anhydrous dichloromethane (5 mL) and the mixture was stirred at room temperature for 5 min. After **11** and HOBt (90 mg, 0.66 mmol) were added to the mixture, DCC (138 mg, 0.66 mmol) in anhydrous dichloromethane (2 mL) was added dropwise to the solution at 0 °C. The mixture was stirred for 30 min at 0 °C and then overnight at room temperature to give a white precipitate which was removed by filtration. The organic phase was washed with saturated aqueous sodium bicarbonate, brine and dried over anhydrous sodium sulfate. After filtered and concentrated, the crude product was purified by column chromatography (petroleum ether/ethyl acetate = 1:3) to afford the desired compound **13** (yield: 60.5%). m.p. 188 °C; IR (cm^−1^): ν 3358, 3326, 2928, 2850, 1625, 1574, 1535, 1307, 1247, 1088; ^1^H-NMR (DMSO-*d*_6_) δ (ppm): 8.38 (1H, s, 2-H), 8.14 (1H, m, -NH), 8.09 (1H, m, -NH), 8.02–8.03 (1H, m, -NH), 7.54 (1H, s, 5-H), 7.24–7.26 (15H, m, Ar), 7.08 (1H, s, 8-H), 3.89 (3H, s, -OCH_3_), 3.86 (3H, s, -OCH_3_), 3.63(2H, m, -CH_2_), 3.52-3.59 (4H, m, -CH_2_), 2.86 (2H, s, -CH_2_); ^13^C-NMR (DMSO-*d*_6_) δ (ppm): 168.87 (C=O), 167.61 (C=O), 158.39 (7-C), 156.59 (4-C), 153.92 (2-C), 152.91 (6-C), 148.41 (9-C), 143.99 (3Ar-C), 129.03 (6Ar-C), 128.04 (6Ar-C), 126.77 (3Ar-C), 108.26 (10-C), 106.21 (8-C), 102.02 (5-C), 65.92 (-CH), 55.94 (-OCH_3_), 55.71 (-OCH_3_), 47.46 (-CH_2_), 42.35 (-CH_2_), 37.96 (-CH_2_), 35.89 (-CH_2_); MS (ESI): M = 621.2 (Found: 622.2); Anal. Calcd. for C_35_H_35_N_5_O_4_S: C, 67.61; H, 5.67; N, 11.26%. Found: C, 67.58; H, 5.72; N, 11.39%.

*Conjugation of Tr-MAG_3_ and ADMQ* (Tr-MAG_3_-ADMQ,**14**). Compound **14** was using the same procedure as for the synthesis of **13** except using Tr-MAG_3_ instead of Tr-MAG (yield: 60.1%). m.p.: 210 °C; IR (cm^−1^): ν 3428, 3290, 1632, 1506, 1247, 1221, 704; ^1^H-NMR (DMSO-*d*_6_) δ (ppm): 8.42 (1H, s, 2-H), 8.34 (1H, m, -NH), 8.19 (1H, m, -NH), 8.17 (1H, m, -NH), 8.11 (1H, m, -NH), 7.99 (1H, m, -NH), 7.62 (1H, s, 5-H), 7.24–7.26 (15H, m, Ar), 7.08 (1H, s, 8-H), 3.89 (6H, s, -OCH_3_), 3.58-3.88 (10H, m, -CH_2_), 2.85 (2H, s, -CH_2_); ^13^C-NMR (DMSO-*d*_6_) δ (ppm): 169.13 (C=O), 169.09 (C=O), 167.77 (2C=O), 158.34 (4, 7-C), 153.85 (2-C), 153.10 (6-C), 148.36 (9-C), 143.95 (3Ar-C), 129.01 (6Ar-C), 128.03 (6Ar-C), 126.75 (3Ar-C), 108.35 (10-C), 106.47 (8-C), 102.01 (5-C), 65.92 (-CH), 55.92 (-OCH_3_), 55.64 (-OCH_3_), 42.31 (2CH_2_), 42.05 (2CH_2_), 38.02 (-CH_2_), 35.85 (-CH_2_); MS (ESI): M = 735.3 (Found: 736.3); Anal. Calcd. for C_39_H_41_N_7_O_6_S: C, 63.66; H, 5.62; N, 13.32%. Found: C, 63.58; H, 5.71; N, 13.39%.

### Synthesis of ^99m^TcN-MAG-ADMQ (**[^99m^TcN]-1**)

Compound **13** (5 mg) was treated with trifluoroacetic acid under cation trapping conditions (5% triethylsilane) at room temperature for 5 min and then the solvent was removed under a stream of nitrogen to give a residue which was neutralized with 0.1 M NaOH. The solution was extracted with dichloromethane for three times, and then the aqueous phase was placed under nitrogen protection. Saline (1 mL) containing [^99m^TcO_4_]^−^ (15 MBq) was added to a kit containing stannous chloride dihydrate (0.05 mg), succinic dihydrazide (SDH, 5.0 mg) and propylenediamine tetraacetic acid (PDTA, 5.0 mg). The mixture was kept at room temperature for 15 min and then the MAG-ADMQ solution (1 mL) was added. The reaction mixture was allowed to stand for 15 min at 100 °C to give the final complex ^99m^TcN-MAG-ADMQ (**[^99m^TcN]-1**).

### Synthesis of ^99m^TcN-MAG_3_- ADMQ (**[^99m^TcN]-2**)

**[^99m^TcN]-2** was prepared using the same method as **[^99m^TcN]-1** except using compound **14** instead of compound **13**.

### 3.3. In Vitro Stability Study

Stability of the two complexes was studied using radio-HPLC analysis at different time intervals as soon as they were prepared. After the complexes were added to test tubes containing PBS solution (0.025 M, pH 7.4), the mixtures were incubated at 37 °C by shaking in a thermomixer. The radiochemical purity was measured at 30 min, 60 min and 120 min.

### 3.4. Radio-HPLC Analysis

The formation of the two complexes was routinely determined by Radio-HPLC. Water (solvent A) and acetonitrile (solvent B) were used for elution. For analysis of the product, the HPLC gradient system started with 100% A/0% B with a linear gradient to 0% A/100% B from 0 to 30 min. The flow rate was 1.0 mL/min. Sample (5 μL) was used for analysis. Recovery was determined by summing the total counts in all fractions and comparing them to the total injected activity.

### 3.5. Paper Electrophoresis

A 1 µL sample was spotted on a piece of Whatman 1 chromatography paper (length 15 cm) which was saturated with 0.05 M pH 7.4 phosphate buffer in an electrophoresis bath in advance. 150 V was applied for 2 h. The strip was then dried and divided into three segments. The distribution of radioactivity on each strip was determined.

### 3.6. Measurement of Partition Coefficients

The two complexes were each mixed with an equal volume of 1-octanol and phosphate buffer (0.025 M, pH 7.4) in a centrifuge tube. After vortexing at room temperature for 1 min, the mixture was centrifuged at 5,000 r/min for 5 min. The aliquot of each phase (0.1 mL) was pipetted and counted in a well-type NaI(Tl) detector and the process was repeated three times. The partition coefficient value was usually expressed as log P, where the partition coefficient, P, was calculated using the following equation:

P = (cps in octanol-cps in background)/(cps in buffer-cps in background)

### 3.7. Biodistribution Study

Biodistribution studies of the two complexes were performed in Kunming female mice (18–22 g) bearing S 180 tumors, which grew to a leg diameter of 10–15 mm. ^99m^TcN-MAG-ADMQ, and ^99m^TcN-MAG_3_-ADMQ (0.1 mL, 7.4 × 105 Bq) were injected via the tail vein and the injected radioactivity was measured with a well-type NaI(Tl) detector, respectively. The mice were sacrificed at 30 min, 60 min and 120 min post-injection and the tumor, other organs of interest and blood were collected, weighed and measured for radioactivity. The results were expressed as the percent uptake of injected dose per gram of tissue (%ID/g). All biodistribution studies were carried out in compliance with the national laws related to the conduct of animal experimentation.

## 4. Conclusions

In summary, 4-(2-aminoethylamino)-6,7-dimethoxyquinazoline (ADMQ) was successfully synthesized and conjugated with MAG and MAG_3_, respectively. The two new compounds were labeled with technetium-99m in high yields through a ligand exchange reaction, which was easily used for the preparation of a radiopharmaceutical through a freeze-dried kit formulation. Moreover, **[^99m^TcN]-2** demonstrates tumor accumulation, high tumor-to-muscle (T/M) ratios and rapid washout from blood. Thus, it appears of high interest to explore the **[^99m^TcN]-2** as a potential single photon emission computed tomography (SPECT) tumor imaging agent.
